# Reliability of Chest Wall Perforator Flaps for Breast Reshaping following Massive Weight Loss

**DOI:** 10.1007/s00266-024-04344-z

**Published:** 2024-09-19

**Authors:** Mai Raafat Abdelazim Hammad, Mohamed Samir Badawy, Eman Nagy Naguib, Amr Mabrouk

**Affiliations:** Department of Plastic, Burn and Maxillofacial Surgery, Ain Shams University, 54 Abdullah Ebn Taher street, Nasr City, Cairo, 11731 Egypt

**Keywords:** Massive weight loss, Breast, Mammography, Mastopexy, Augmentation mastopexy

## Abstract

**Background:**

Breast deformity following massive weight loss poses a unique challenge inadequately managed by traditional methods. Patients also have considerable lateral and posterior upper trunk tissue surplus.

Multiple studies have used chest wall flaps for auto-augmentation with mastopexy to tackle this problem. However, the outcome measures did not include any objective tools to assess the reliability of these flaps. Hence, in this study sono-mammography and breast anthropometric measurements are used for evaluation of the added volume and long-term sustainability of chest wall perforator flaps.

**Methodology:**

Twenty massive weight loss patients with Pittsburgh Rating Scale score 2-3 underwent mastopexy with autologous augmentation through perforator flaps encompassing lateral chest wall skin. Outcome measures were breast volume, and the presence of fat necrosis on mammography, and direct breast anthropometry.

**Conclusion:**

All patients showed a consistent increase in volume postoperatively and no fat necrosis in postoperative mammography, reflecting flap reliability over one year of follow-up. All postoperative anthropometric measurements showed improvement in the breast deformities addressed.

**Level of Evidence IV:**

This journal requires that authors assign a level of evidence to each article. For a full description of these Evidence-Based Medicine ratings, please refer to the Table of Contents or the online Instructions to Authors  www.springer.com/00266.

## Introduction

Managing the breast after massive weight loss (MWL) poses unique challenges that are often inadequately managed by traditional techniques [1]. Deflation, vertical and horizontal ptosis, medialization of the nipple areolar complex (NAC), loss of the lateral curvature of the breast, and involvement of an axillary tissue roll are some of the characteristics of the breasts following MWL [1, 2]. In addition, patients have considerable lateral and posterior tissue redundancy of the upper trunk, necessitating an upper body dermo-lipectomy for the treatment of “back rolls” [3].

Chest wall perforator flaps were originally used for partial breast reconstruction following tumor excision [4] but multiple studies have used them for auto-augmentation with mastopexy in MWL patients, taking advantage of these patients’ considerable lateral and posterior tissue excess of the upper trunk [3, 5–8].

The outcome measures for these studies were patient satisfaction [8], need for revision surgery [3], and the development of complications [7]. However, none of these studies used any objective tools to assess the reliability of these “buried” flaps, or assess the added breast volume, through the evaluation of their long-term reliability or aesthetic outcome, which was thus highlighted in this study using sono-mammography and breast anthropometric measurements. Mammography was chosen for as it is the most studied and preferred imaging modality for evaluating fat necrosis of the breast [9].

Thus, the aim for this study was to use sono-mammography for evaluation of the reliability, added volume and long-term sustainability of chest wall perforator flaps when used for breast auto-augmentation following MWL and to assess the aesthetic outcomes.

### Patients and Methods

This prospective clinical study was done over a two-year period from July 2021 to July 2023 in the Plastic Surgery Department of Ain Shams University hospitals, Cairo, Egypt. Local Ethical Committee approval and patient informed written consent to participate in the study. Twenty patients presenting to Ain Shams University Hospitals were enrolled.

### Inclusion Criteria

Women aged 18–45 years old, who had lost ≥ 50% of excess weight following surgical or non-surgical interventions (MWL), with stable weight for at least 6 months (to achieve metabolic and nutritional homeostasis and decrease the risk of surgical complications) [10], and BMI range 18.5–30 kg/m^2^.

Patients who had ptotic breasts with Pittsburgh Rating Scale (PRS) score 2–3 [11] seek autologous augmentation or refuse implants and require simultaneous contouring of lateral chest wall redundancy.

### Exclusion Criteria

Patients were excluded if they had medical comorbidities (e.g., uncontrolled diabetes, uncontrolled hypertension, thyroid diseases, hormonal disturbances, and cardiopulmonary diseases), hereditary conditions that affect wound healing (e.g., Ehler Danlos Syndrome, and Progeria), were active smokers (Smokers are required to stop smoking 1 month before surgery), underwent previous breast surgery, and had congenital breast deformity (e.g., tuberous breasts) and breast masses. Pregnant or lactating patients were also excluded.

### Local Examination

Patients’ breasts were examined for degree of ptosis (Regnault classification), skin laxity, parenchyma, and PRS grade [11]. Direct breast anthropometry measurements were recorded as described by Quieregatto [12] (Sternal notch to nipple, nipple to inframammary fold, and nipple to nipple).

### Investigations

Preoperative sono-mammography was done to estimate breast volume as demonstrated by Kalben [13] and exclude tumors, cysts, or fat necrosis [9].

### Marking

**Fig. 1:**
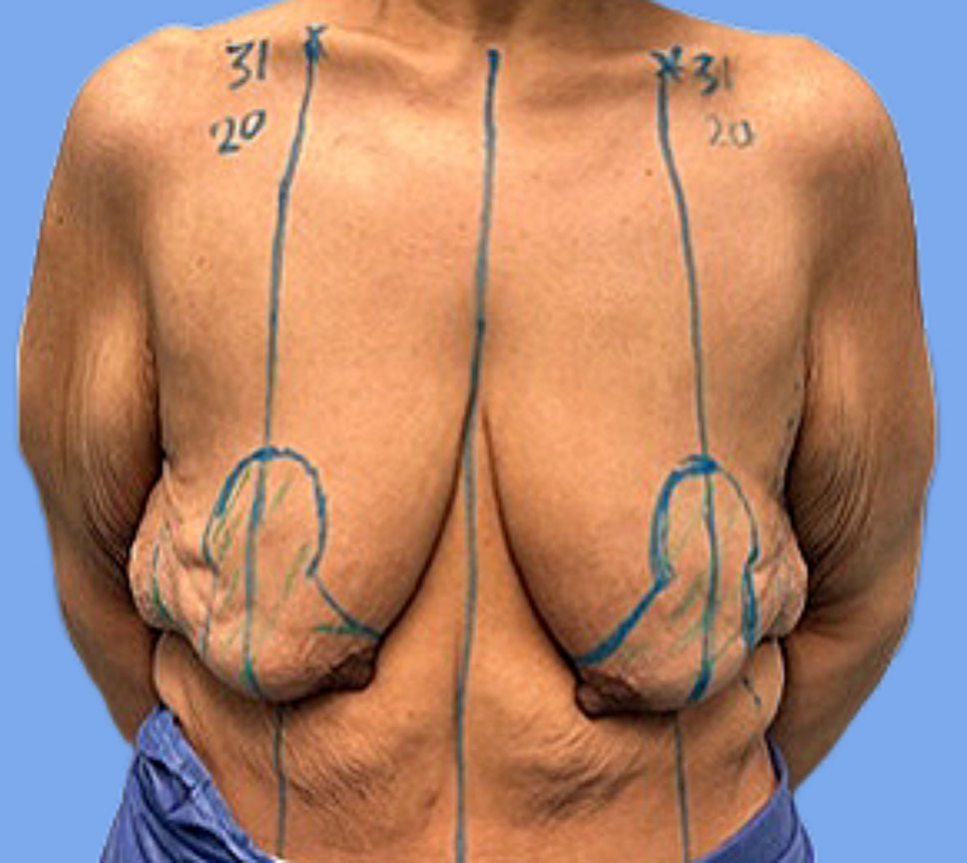
Marking of Wise Pattern incisions (Preoperative SN-N 31 above, target postoperative SN-N 20 below)

Marking was done preoperatively in the standing position for Wise Pattern incisions with preservation of inferior and central pedicles. The desired breast meridian was outlined following the ideal position of the NAC on the breast mound irrespective of what the actual location of the nipple is. The center of the new nipple position was estimated by tracing a horizontal line 1  cm over the inframammary fold (IMF) transposition and then marked on the breast mound at the level of the breast meridian. The superior border of the areola was outlined approximately 2  cm over this marking, setting the areolar diameter at 4 cm. Two vertical lines 7  cm long were traced caudally on each side, extending from the lower limit of the new areolar opening with an angle of 70°. The pedicle was marked from the IMF passing around the NAC. The inferior marking passes along the IMF (Fig. 1).

**Fig. 2:**
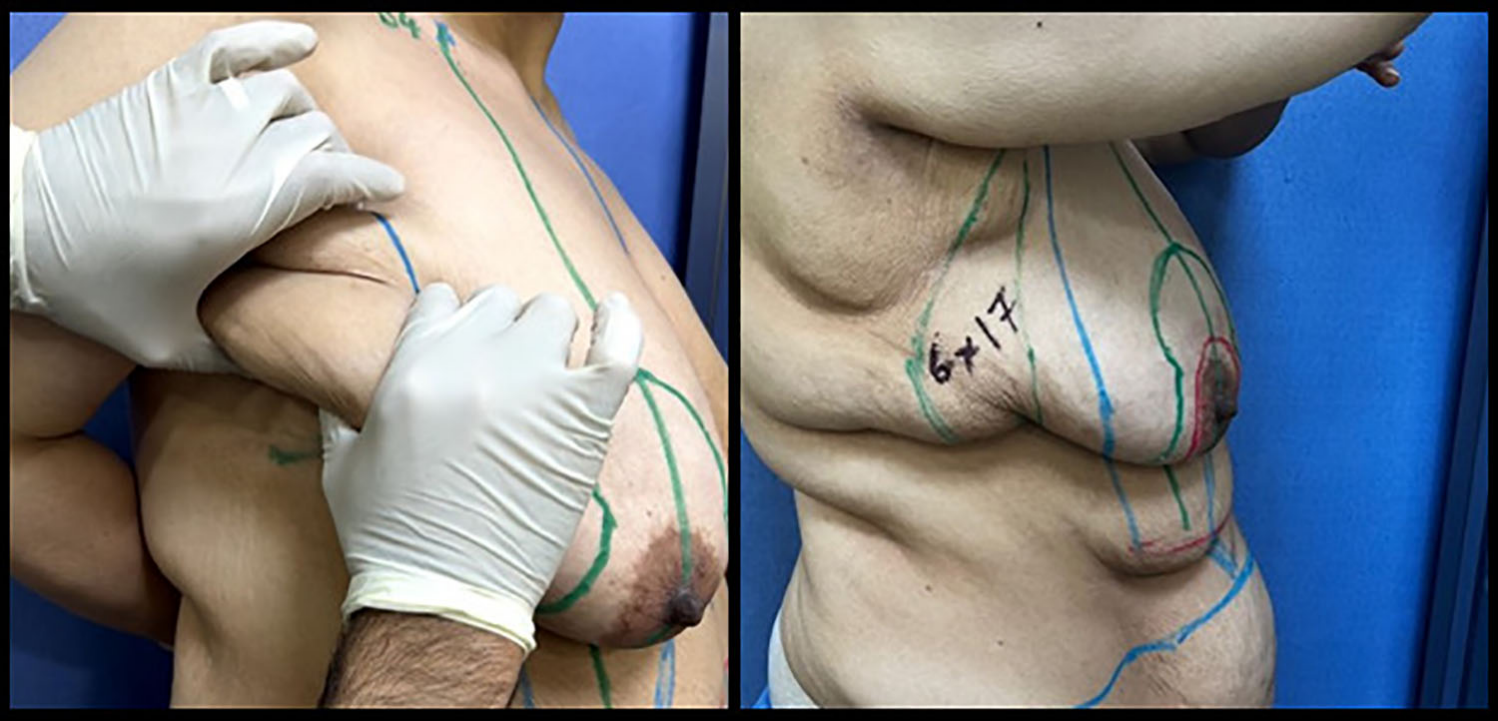
Pinch test to determine the amount of excess tissue (left). Flap dimensions marked (right)

**Fig. 3:**
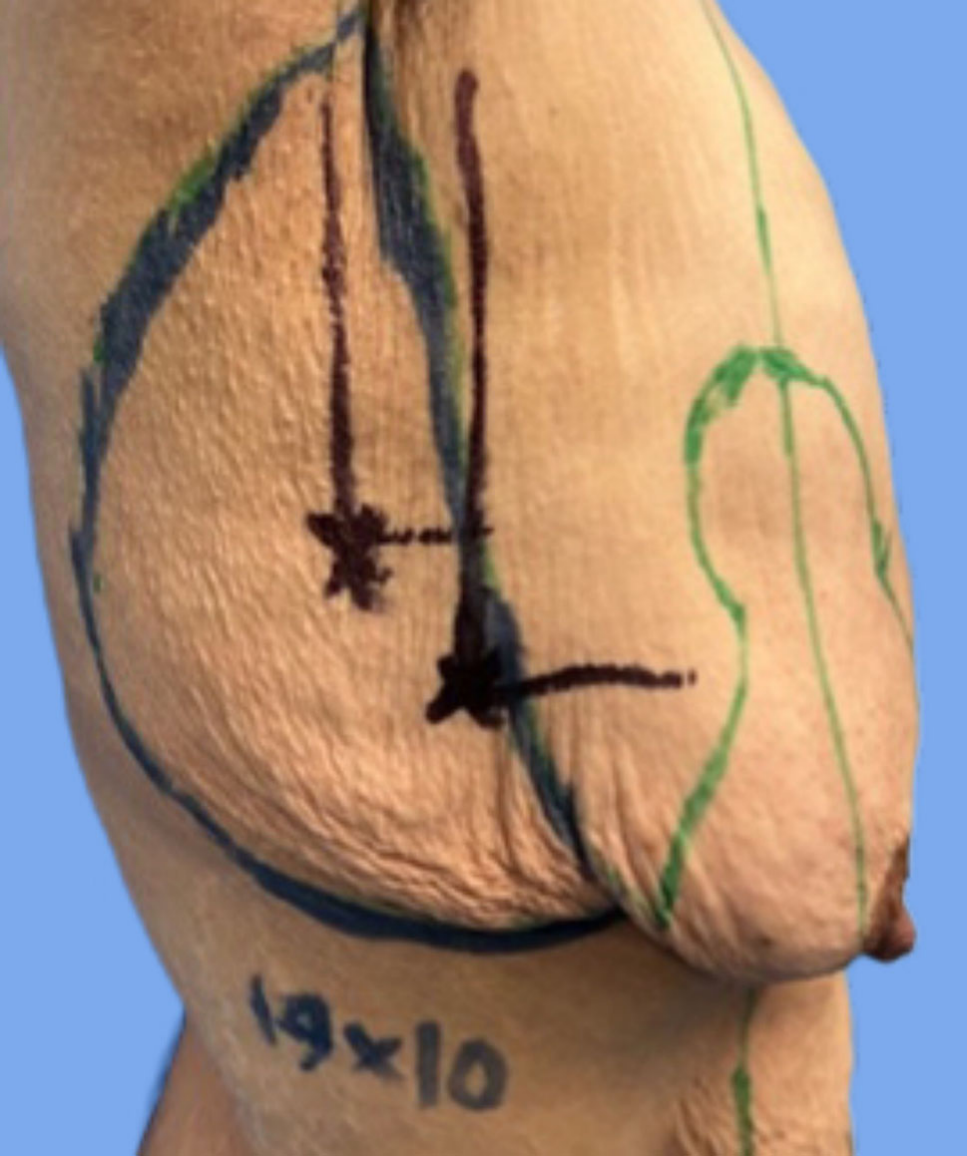
Marking of chest wall perforators

Pinch test was done to determine and mark the amount of tissue excess in the lateral chest wall and hence flap dimensions, as demonstrated in Fig. 2, followed by mapping of the perforators (thoracodorsal artery perforators versus intercostal artery perforators) by a handheld Doppler probe (Fig. 3), which were also marked.

### Surgical Technique

**Fig. 4:**
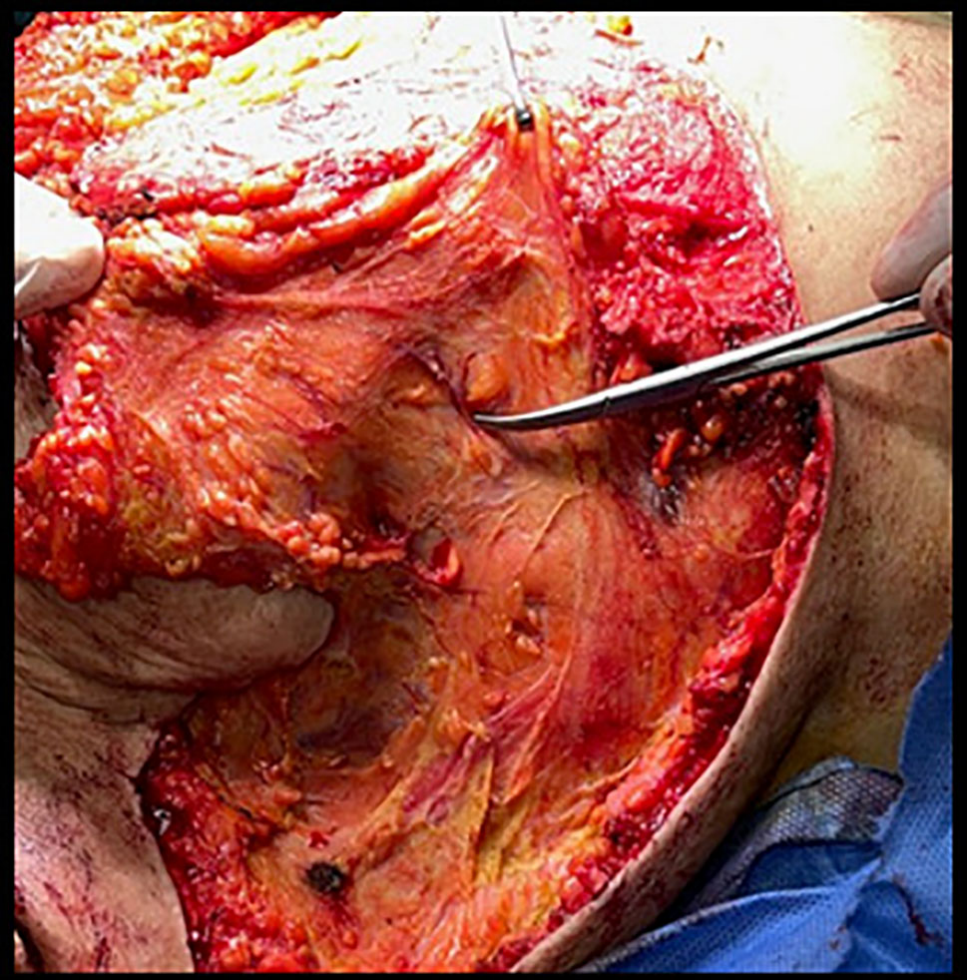
Intraoperative perforators

**Fig. 5:**
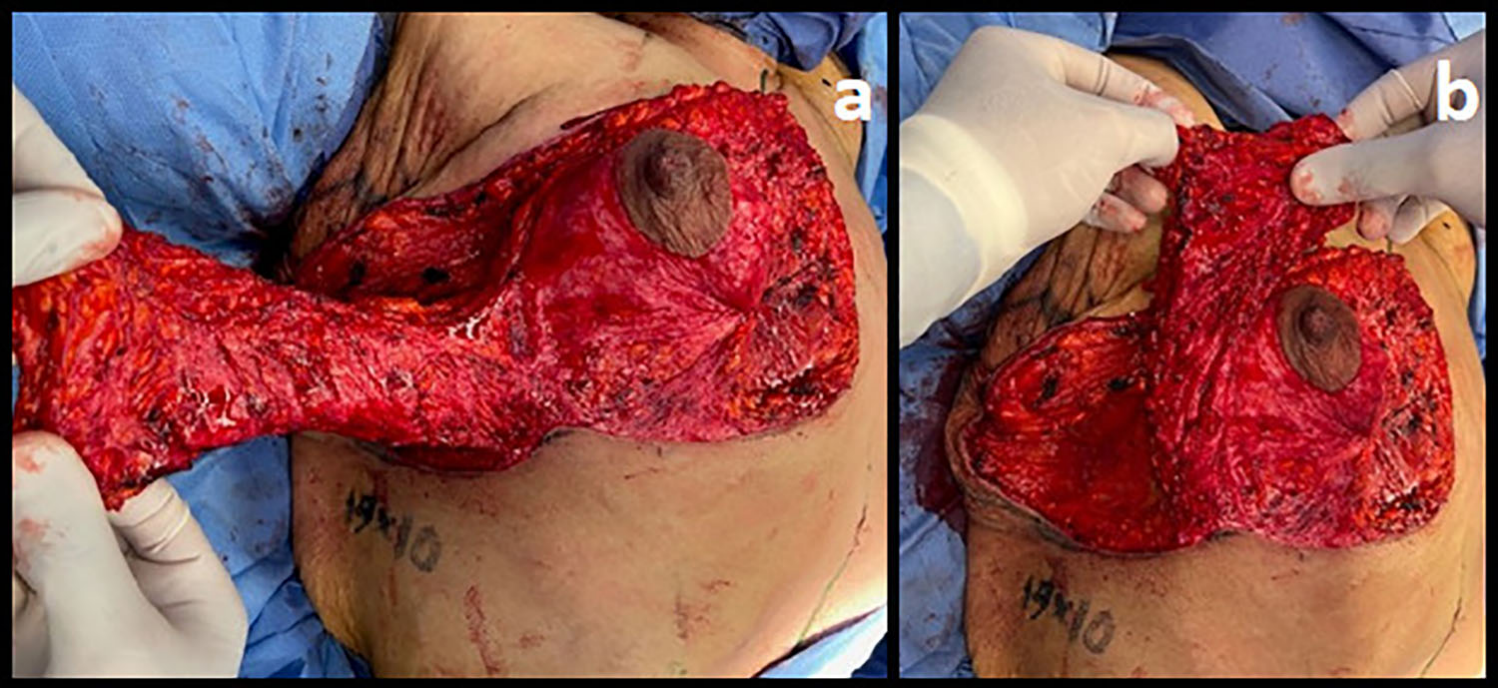
De-epithelialized lateral chest wall flap (**a**). Transposition of flap to the upper pole (**b**)

Patients received general anesthesia and were positioned supine. Mastopexy with auto-augmentation as described by Rubin and Khachi [14], was performed, preserving an inferior and central pedicle, in addition to a lateral extension to encompass the lateral chest wall skin roll that was previously estimated **(**Fig. 3). We also added an extended brachioplasty when indicated. Intraoperative perforators were marked (Fig. 4) and then the flap encompassing the lateral chest wall was de-epithelialized (Fig. 5a) and transposed to the upper breast pole (Fig. 5b) and anchored by permanent sutures to the second rib periosteum. Surgical drains were placed in the breast and donor site preceding skin closure when needed. Drain was removed according to amount and color (less than 50 cc/ serous). Follow-up visits were at postoperative day 7, 10, 14, and 21 to detect any complications such as seroma, wound dehiscence, infection (redness, hotness, tenderness, and discharge).

### Outcome Measures

At 6 months and 1 year postoperatively, sono-mammography was done to measure breast volume and to detect signs of fat necrosis which would reflect partial or total flap loss. To reflect the aesthetic outcome, direct breast anthropometry measurements were repeated.

### Statistical Analysis

The results are presented as number and percentage for qualitative data or mean and standard deviation for quantitative data. Two sets of quantitative data were compared using Student’s t-test for dependent or independent means as appropriate. One-way analysis of variance (ANOVA) was used in comparing three sets of quantitative data. The results were considered statistically significant if *P* value was below 0.05.

Statistical analysis was performed using computer software statistical package for the social science (SPSS, version 20; SPSS Inc., Chicago, Illinois, USA)

## Results

### Patient Characteristics

Mean age was 33.4±7, with a range of 20–42 years. Previous weight was 142.7±29.5 kilograms (kg). Preoperative weight was 64.65±7 kg. Mean body mass index (BMI) was 24.9±2.75.

## Anthropometric measurements

Postoperative sternal notch to nipple (SN-N), nipple to IMF (N-IMF), and nipple to nipple measurements (N-N) were compared to preoperative measurements and then compared after 6 months and 1 year.

Mean preoperative SN-N was 32.1±3.5 cm, while postoperative SN-N was 23.25±2.7. Mean postoperative SN-N after 6 months was 23.5±2.3, and 23.7±2.1 after 1 year showing a difference that is not statistically significant from the immediate postoperative results.

Mean preoperative N-IMF was 12.6±2, decreasing to 9.85±1.9 postoperatively, and showing a statistically insignificant increase over the follow-up period (10.15±1.8 at 6 months, and 10.25±1.7 at 1 year).

Mean preoperative N-N was 16.35±2.5, increasing postoperatively to 20.45±2. There was a nonsignificant increase at 6 months and 1 year postoperatively.

Anthropometric measurements are summarized in Table 1.

**Table 1:** Anthropometric measurements mean (standard deviation)

SN-N preoperative	SN-N postoperative	*t* value	*p* value	
32.1 (3.53)	23.15 (2.7)	8.969	<.00001	
SN-N postoperative	SN-N 6-month postoperative	SN-N 1-year postoperative	*F* value	*p* value
23.15 (2.7)	23.5 (2.3)	23.7 (2)	0.26957	0.76468
N-IMF preoperative	N-IMF postoperative	*t* value	*p* value	
12.6 (2.1)	9.85 (1.9)	4.30099	0.000114	
N-IMF postoperative	N-IMF 6-month postoperative	N-IMF 1-year postoperative	*F* value	*p* value
9.85 (1.9)	10.15 (1.8)	10.25 (1.7)	0.25616	0.774906
N-N preoperative	N-N 6-month postoperative	*t* value	*p* value	
16.35 (2.5)	20.45 (1.96)	− 5.77552	< .00001	
N-N postoperative	N-N 6-month postoperative	N-N 1-year postoperative	*F* value	*p* value
20.45 (1.96)	20.7 (2)	20.65 (2)	0.08762	0.916236

**Fig. 6:**
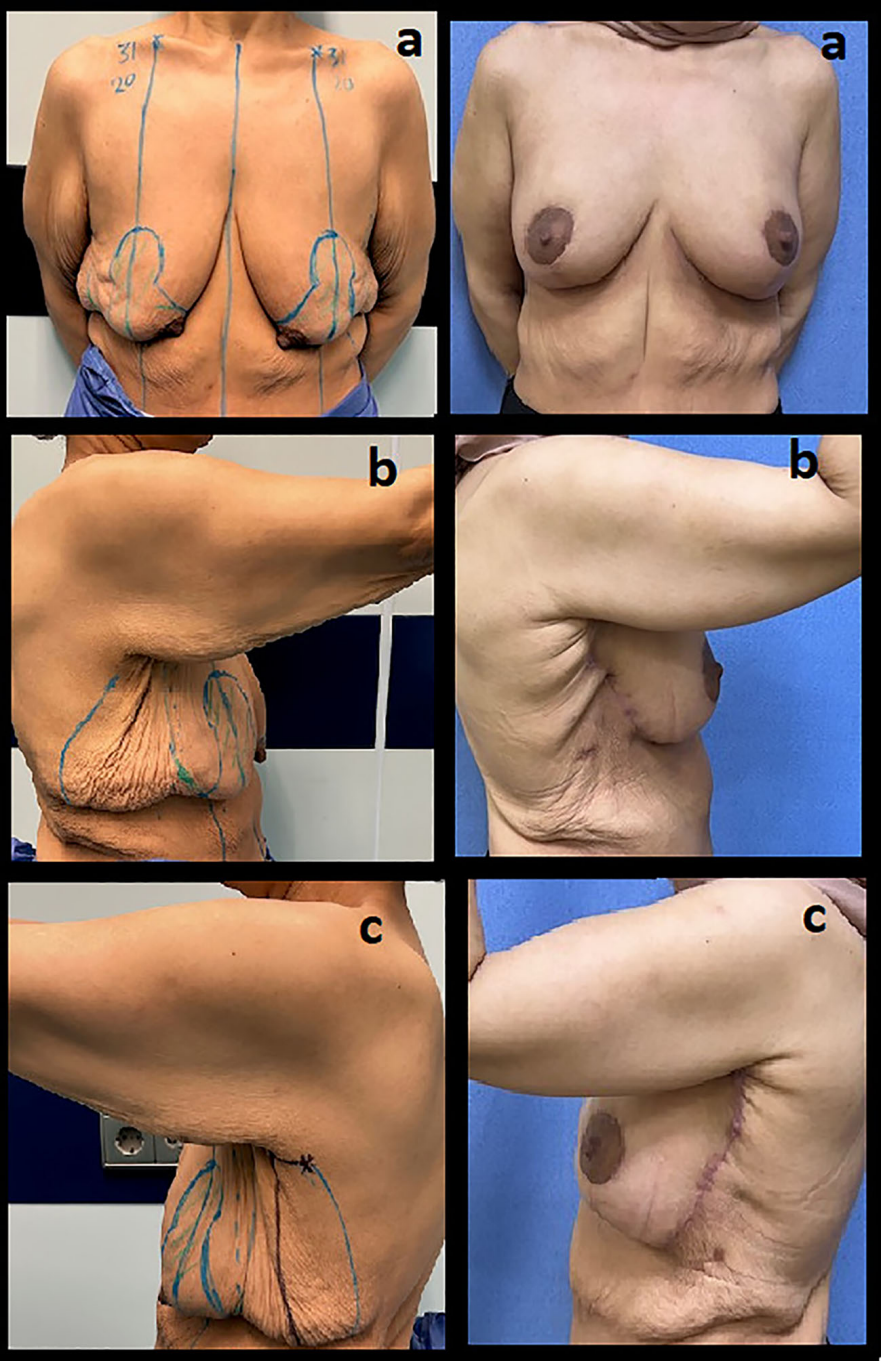
A 37-year-old patient. Preoperative photographs (Left: **a**: Frontal view, **b**: Right lateral view, **c**: Left lateral view). postoperative photographs at 6 months (Right: **a**: Frontal view, **b**: Right lateral view, **c**: Left lateral view)

**Fig. 7:**
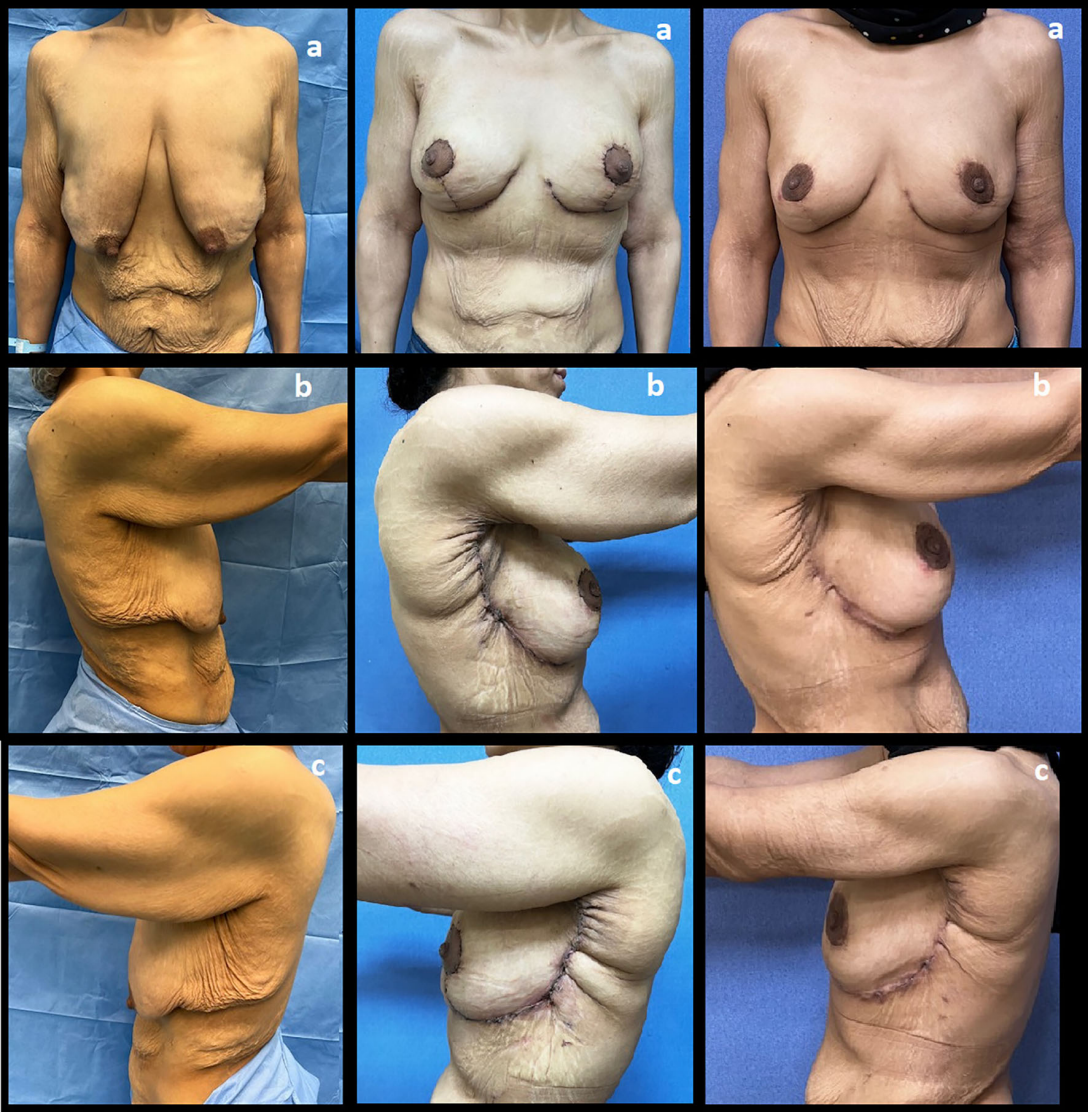
A 36-year-old patient. Preoperative photographs (Left: **a**: Frontal view, **b**: Right lateral view, **c**: Left lateral view). postoperative photographs at 1 month (Middle: **a**: Frontal view, **b**: Right lateral view, **c**: Left lateral view). postoperative photographs at 3 months (Right: **a**: Frontal view, **b**: Right lateral view, **c**: Left lateral view)

Figures 6 and 7 show the preoperative and postoperative photographs for two patients in this study.

### Sono-mammography

Regarding breast volume measurements, the mean preoperative value was 410±129 mL, increasing to 657±129 mL at 6 months postoperatively, and then dropping nonsignificantly to 649.6±127 mL at 1 year.

**Fig. 8:**
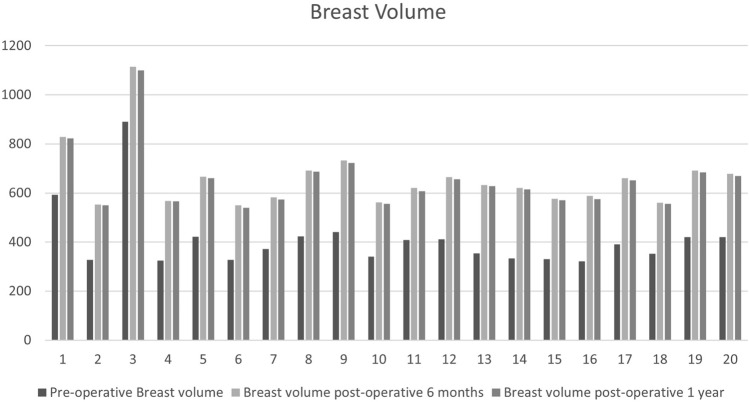
Preoperative and postoperative breast volume measurements

Flap volume was also estimated, being 247±26 and 239±27 mL at 6-month and one-year postoperative, respectively. Breast volume and flap volume measurements are summarized in Table 2. Preoperative and postoperative breast volume for all patients are demonstrated in Fig. 8. Preoperative sono-mammography for all patients showed no abnormalities, except for one patient with BIRADS I fibroadenosis [15] and another patient who has a BIRADS III mass, from which a core biopsy was histopathologically examined showing a benign fibroma. At 6 months and one year postoperatively, all patients showed no oil cysts (fat necrosis representing partial or total flap loss) reflecting flap reliability over the long term.

**Table 2:** Breast volume and flap volume mean (Standard deviation)

Preoperative breast volume	Postoperative breast volume at 6 months	*t* value	*p* value	
410.25 (129)	657.4 (129)	− 6.04425	< .00001	
Postoperative breast volume at 6 months	postoperative breast volume at 1 year	*t* value	*p* value	
657.4 (129)	649.65 (127.5)	0.19109	0.84947	
Flap volume at 6 months	Flap volume at 1 year	*t* value	*p* value	
247.15 (26.4)	239.4 (26.8)	0.92052	0.36310

### Complications

**Fig. 9:**
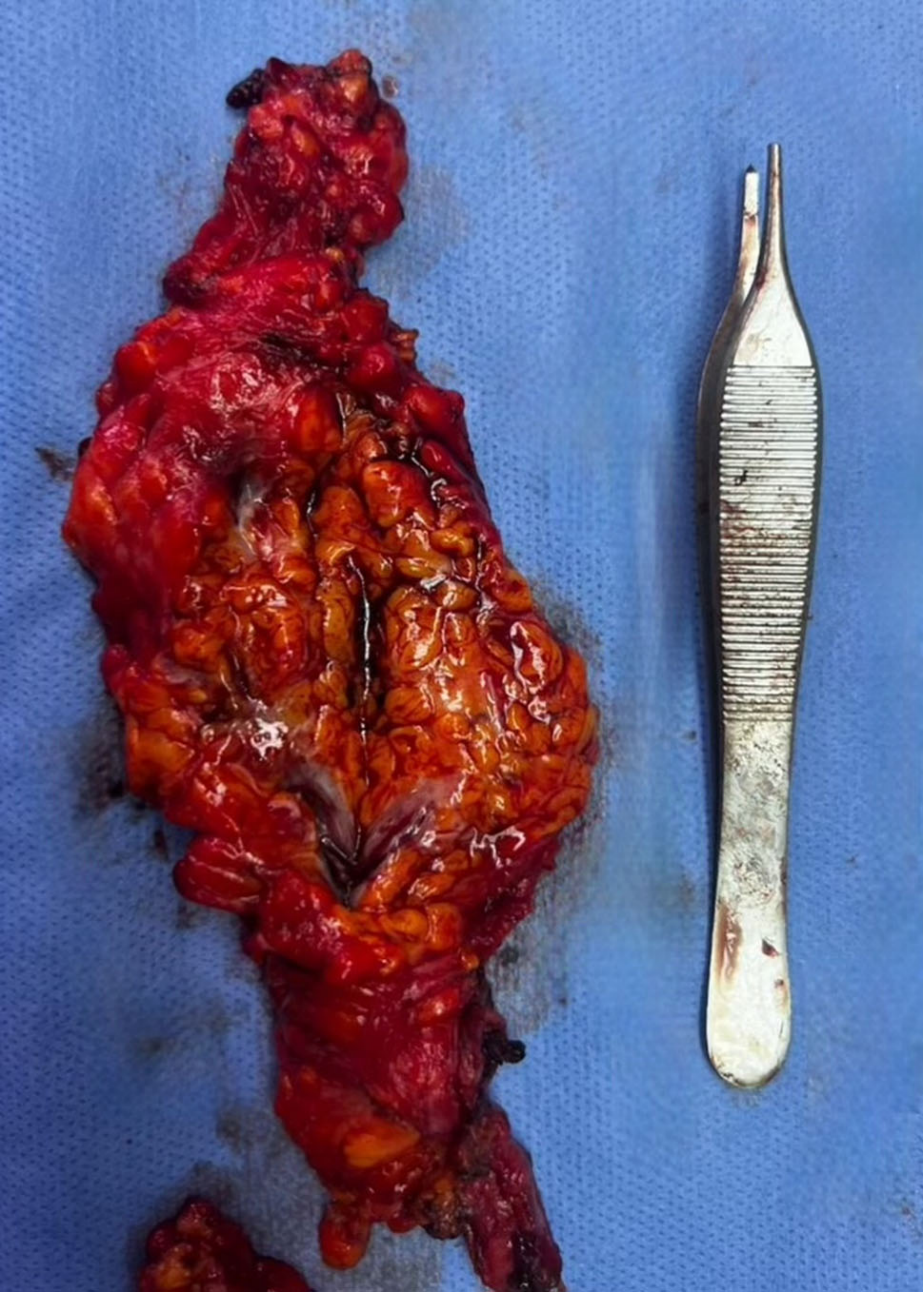
Encysted seroma from left arm of a patient who underwent simultaneous brachioplasty (excised on day 36)

Only three patients had complications. One had a wound disruption at the left axilla at the junction between the mastopexy and brachioplasty wounds (1x2 cm) which healed after three further weeks of dressings. One patient developed a seroma in her right arm on postoperative day 10, which was managed conservatively by aspiration and compression garments. One patient had an encysted seroma in her left arm, which developed on postoperative day 14, serially aspirated on day 14–21 and 28, then surgically excised on day 36 (Fig. 9).

## Discussion

Following MWL, patients typically present with skin redundancy, which causes enormous aesthetic, physical, and psychological problems. Body contouring after MWL is now embraced as a safe and reliable method to improve self-esteem, social life, work ability, and physical and sexual activity [16].

The breasts are often part of this complex deformity following MWL, which varies depending on BMI, breast size, and amount of weight loss. This includes both vertical and horizontal ptosis, inferiorly displaced inframammary fold (IMF), loss of upper pole volume, medialization and distortion of the NAC, and extreme skin laxity and inelasticity [1]. In addition, patients have considerable lateral and posterior tissue surplus of the upper trunk [3].

Most techniques described for the reshaping of the MWL breast include one or more of the following principles: parenchymal plication, dermal suspension, and auto-augmentation. These are done to prevent or minimize recurrence, which is a characteristic problem in MWL patients [1, 17].

Chest wall perforator flaps were originally used for partial breast reconstruction following tumor Multiple studies have used chest wall perforator flaps for auto-augmentation with mastopexy in MWL patients [3, 5–8]

In our study, patients underwent auto-augmentation using pedicled lateral chest wall flaps from the excess lateral skin roll. Perforators were marked preoperatively and intraoperatively the flap encompassing the lateral chest skin roll was de-epithelialized and transposed to the upper breast pole and fixed by permanent sutures to the second rib periosteum.

The outcome measures for previous studies were patient satisfaction [8], need for revision surgery [3], and the development of complications [7]. However, none used any objective tools to assess the flaps or the added breast volume.

In our study, this was highlighted using sono-mammography and breast anthropometric measurements. All postoperative anthropometric measurements showed improvement in the breast deformities needed to be addressed in a MWL patient, and consistent results over the follow-up period. All patients showed a consistent increase in volume postoperatively and no fat necrosis in their postoperative sono-mammography, reflecting flap reliability over 1 year.

Numerous techniques use dermoglandular and fasciocutaneous flaps to improve volume and shape, avoiding the need for implant augmentation. Hurwitz and Agha-Mohammadi described a technique using the excess tissue coming from the epigastrium and the lateral thoracic region and creating a double-pedicle (one medial and one lateral) auto-prosthesis to fill and give projection to the breast mound. However, this technique offered better results when combined with a reverse abdominoplasty and often required a second operative time with a prosthetic implant [18]. They also reported an incidence of necrosis of the apical portion of the flaps in up to 20% of cases [19], probably because of the considerable length of the flaps. The use of superior abdominal excess during a reverse abdominoplasty has also been described in which the excess tissue is de-epithelialized and reshaped as an auto-prosthesis to improve volume and projection. However, with this technique the risk of cancel the inframammary fold (IMF) is high because moving the flap from the upper abdomen to the breast, the pedicle had to cross the IMF with the risk of reducing its definition [20].

The use of remote flaps taken from the abdomen (transverse rectus abdominis myocutaneous flap or deep inferior epigastric perforator flap) and from the inner thigh or gluteal region are technically more demanding and require microsurgical knowledge. In addition, these techniques are more invasive, often require longer operative time, and are not feasible if the patients previously underwent upper body lift, brachioplasty, or abdominoplasty.

Hamdi [4] utilized pedicled perforator flaps in partial breast reconstruction, namely the thoracodorsal artery perforator (TDAP) flap and the intercostal artery perforator (ICAP) flap using a clinical algorithm based on the location of the defect and the availability of these perforators*.* A case report of pedicled perforator flaps for breast augmentation was subsequently published [21].

Since then, multiple studies have used chest wall perforator flaps for auto-augmentation with mastopexy in MWL patients, making use of their lateral tissue excess while simultaneously improving the contour of this area [3, 5–8].

Incorporation of perforators into the flaps, preservation of the subdermal plexus, and adherence to appropriate length-to-width ratios allows for reliable transfer, with minimal potential for fat necrosis or flap loss, as opposed to random tissue transfer. [1] Perforator dissection is not necessary if the flap can easily reach the desired subglandular pocket; however, it significantly increases the arc of rotation beyond the lateral breast, allowing rotation of the flap into the superomedial portions of the breast, which are often precisely the areas of the breast most in need of augmentation following MWL [1].

We found a limitation in the number of patients seeking body contouring in our institution following MWL which we attribute to the lack of patient education regarding the availability of body contouring surgeries following MWL.

Further research is needed to compare different methods of auto-augmentation regarding volume of tissue, especially in comparison with implant augmentation.
